# A147 CHARACTERIZATION OF EPIGENETIC MODIFICATIONS OF HUMAN COLONOID MONOLAYERS ESTABLISHED AS *IN VITRO* CHRONIC DAMAGE MODEL

**DOI:** 10.1093/jcag/gwae059.147

**Published:** 2025-02-10

**Authors:** S Sandilya, K Dever, J Jiang, B Rees, M Kobor, T Steiner

**Affiliations:** Microbiology and Immunology, The University of British Columbia, Vancouver, BC, Canada; Microbiology and Immunology, The University of British Columbia, Vancouver, BC, Canada; Microbiology and Immunology, The University of British Columbia, Vancouver, BC, Canada; Microbiology and Immunology, The University of British Columbia, Vancouver, BC, Canada; Microbiology and Immunology, The University of British Columbia, Vancouver, BC, Canada; Microbiology and Immunology, The University of British Columbia, Vancouver, BC, Canada

## Abstract

**Background:**

Our lab established an *in vitro* damage model using human colonoids grown as 2D Intestinal Epithelial Cells (IECs) monolayers in Air-Liquid Interface (ALI) culture. Upon repeated injury by submergence, these colonoid monolayers lost their epithelial barrier integrity and regrowth potential. Changes in mRNA expression and global proteomic profiling of this human model of injury were found to be very similar to those found in Inflammatory Bowel Disease (IBD) and pre-colon cancer like state.

**Aims:**

Preliminary proteomics studies on these monolayers is suggestive of significant changes in expression of key proteins after progressive rounds of injury. These are quite similar to that seen in IBD. These could be strongly associated with epigenetic modifications, such as, DNA methylation and chromatin accessibility which would impact the gene expression, in these epithelial monolayers.

This study aims to assess the epigenetic modifications of these injured IECs monolayers which will help in identification and validation of methylation at specific sites.

**Methods:**

To assess the epigenetic modifications, these IEC monolayers from 2 human colonoid lines were analyzed pre-ALI, during growth and differentiation in ALI before injury, and after 1st, 3rd and 5th round of injury and recovery.

Global methylation, at each of the conditions was assessed using the Illumina Infinium Methylation EPIC BeadChip, an array based technology, to assess the bisulphite-converted cellular DNA for methylation at CpG sites. Genomic DNA was extracted and bisulfite conversion was done using Zymo EZ DNA methylation kit. Methylation at specific sites has been calculated using the fluorescence intensity of methylated and unmethylated alleles. DNAm age estimator software, HOMER, was used to calculate the epigenetic age.

Open chromatin accessibility for the viable cells was measured using bulk ATAC-Seq, which uses transposon based barcoding to build a library for sequencing of chromatin-accessible genes. Gene ontology process enrichment analysis has been used to identify changes in gene expression pathways at above mentioned stages.

**Results:**

Progressive rounds of submergence injury lead to loss of barrier integrity, strongly correlated with significant genotypic and phenotypic changes.Increased DNA methylation and histone assembly found in our studies are reflective of chronic damage. Epigenetic characterization has helped in identfication of key specific targets which play modify pathways leading to chronic epithelial injury.

**Conclusions:**

Correlative evidence from proteomics profiling and epigenetic characterization of this *in vitro* chronic damage model provides for an enhanced understanding of the initiation and progression of IBD and IBD associated colorectal cancer.

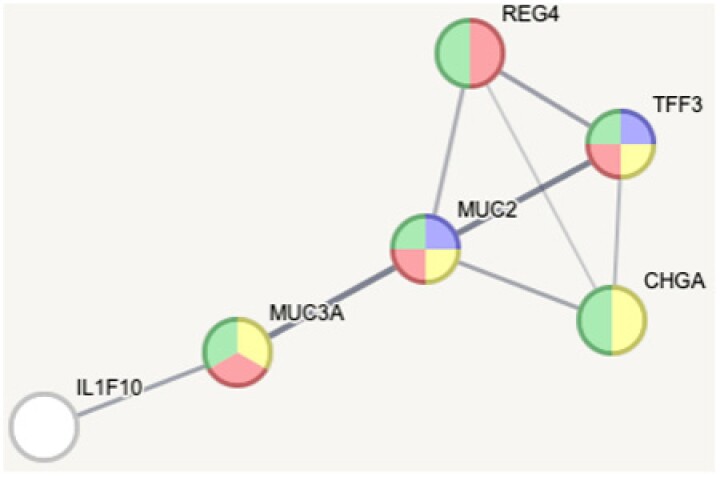

Upregulated proteins after 5th round of damage

**Funding Agencies:**

CCC

